# Transcranial Direct Current Stimulation for Obsessive-Compulsive Disorder: A Systematic Review

**DOI:** 10.3390/brainsci8020037

**Published:** 2018-02-24

**Authors:** Jérôme Brunelin, Marine Mondino, Rémy Bation, Ulrich Palm, Mohamed Saoud, Emmanuel Poulet

**Affiliations:** 1 INSERM, U1028, Lyon Neuroscience Research Center, PSY-R2 team, F-69000 Lyon, France; marine.mondino@ch-le-vinatier.fr (M.M.); remy.bation@chu-lyon.fr (R.B.); mohamed.saoud@chu-lyon.fr (M.S.); emmanuel.poulet@chu-lyon.fr (E.P.); 2 CNRS, UMR5292, Lyon Neuroscience Research Center, PSY-R2 Team, F-69000 Lyon, France; 3 University Lyon, F-69000 Lyon, France; 4 Centre Hospitalier le Vinatier, F-69678 Bron, France; 5 Psychiatry Unit, Wertheimer Hospital, CHU Lyon, F-69500 Bron, France; 6 Department of Psychiatry and Psychotherapy, Klinikum der Universität München, D-80336 Munich, Germany; Ulrich.Palm@med.uni-muenchen.de; 7 Psychiatry Emergency Unit, Edouard Herriot Hospital, CHU, F-69000 Lyon, France

**Keywords:** OCD, tDCS, brain stimulation, neuromodulation, obsession, compulsion

## Abstract

Despite the advances in psychopharmacology and established psychotherapeutic interventions, more than 40% of patients with obsessive-compulsive disorder (OCD) do not respond to conventional treatment approaches. Transcranial direct current stimulation (tDCS) has been recently proposed as a therapeutic tool to alleviate treatment-resistant symptoms in patients with OCD. The aim of this review was to provide a comprehensive overview of the current state of the art and future clinical applications of tDCS in patients with OCD. A literature search conducted on the PubMed database following PRISMA guidelines and completed by a manual search yielded 12 results: eight case reports, three open-label studies (with 5, 8, and 42 participants), and one randomized trial with two active conditions (12 patients). There was no sham-controlled study. A total of 77 patients received active tDCS with a large diversity of electrode montages mainly targeting the dorsolateral prefrontal cortex, the orbitofrontal cortex or the (pre-) supplementary motor area. Despite methodological limitations and the heterogeneity of stimulation parameters, tDCS appears to be a promising tool to decrease obsessive-compulsive symptoms as well as comorbid depression and anxiety in patients with treatment-resistant OCD. Further sham-controlled studies are needed to confirm these preliminary results.

## 1. Introduction

Obsessive-compulsive disorder (OCD) is a frequent and debilitating psychiatric condition that occurs in 2–3% of the population [1]. Symptoms consist of unwanted intrusive thoughts and compulsive behaviours, leading to the inability to maintain social and occupational functioning [2].

Established treatments consist of a combination of psychopharmacology (especially selective serotonin reuptake inhibitor—SSRI) and psychotherapeutic interventions, such as cognitive behavioral therapy—CBT [3]. Despite augmentation strategies with other psychotropic drugs and advances in psychopharmacology [4], it is assumed that nearly 40% of patients do not show a sufficient response to conventional treatments [3]. Therefore, the development of new therapeutic approaches is warranted.

Among the recently developed therapeutic approaches, non-invasive brain stimulation techniques (NIBS), such as transcranial direct current stimulation (tDCS) hold promises to alleviate symptoms and improve cognitive functioning in various psychiatric conditions [5,6]. tDCS consists of applying a weak direct current (1–2 mA) between two electrodes placed on the scalp of a subject. Neurophysiological studies have reported that depending on the electrode polarity and current intensity, tDCS may increase cortical excitability in the vicinity of the anode whereas cathodal tDCS may decrease it [7]. The effects of tDCS are not restricted to the area beneath the electrodes and could reach a widespread network of cortical and subcortical regions that are connected to the targeted region [8]. The ability of tDCS to modulate a network is of particular interest since abnormal activity and connectivity within the orbitofronto-striato-pallido-thalamic network is described in patients with OCD. Indeed, imaging studies in patients with OCD showed abnormalities, which can be either hyper- or hypo activities, within numerous brain regions along a widespread network including the orbitofrontal cortex (OFC), the (pre-) supplementary motor area (SMA), the cingulate gyrus, the caudate, the thalamus, the right and left cerebellum, and the parietal cortex [9]. These abnormalities, which can be either trait- or state-dependent, have been revealed in resting conditions as well as by symptom provocation paradigms, depending on the studies. Moreover, it has been reported that some of these abnormalities were reverted after successful treatment [10,11]. It has thus been hypothesized that applying tDCS over these abnormal brain regions would lead to a decrease in obsessive-compulsive (OC) symptoms by modulating the underlying abnormal brain network. For instance, the use of anodal tDCS over the pre-SMA is based on imaging studies revealing an interaction between pre-SMA hypoactivity and deficient response inhibition with reciprocal striatal hyperactivity in patients with OCD [12]. The use of cathodal tDCS over the OFC is based on imaging studies reporting hyperactivity at rest and during symptom provocation paradigms of the OFC in patients with OCD [9,13]. Targeting the dorsolateral prefrontal cortex (DLPFC) is based on NIBS studies reporting beneficial clinical effects when stimulating this specific brain region in numerous psychiatric conditions [5,6], and on imaging studies reporting abnormalities in the cortico-striato-thalamo-cortical pathways, especially the ‘DLPFC-caudate nucleus-thalamus’ loop that is implicated in the pathophysiology of OCD [13]. This review aimed to provide a comprehensive overview of existing literature on the effects of tDCS applied as a therapeutic tool to reduce OC symptoms in patients with treatment-resistant OCD and to discuss future applications of tDCS in OCD.

## 2. Materials and Methods

### Search Strategy

**Figure 1:**
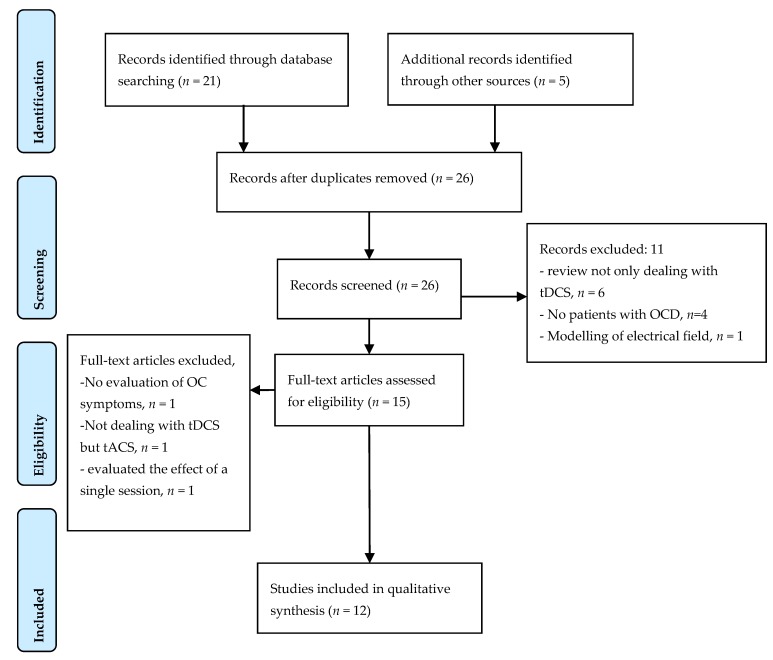
PRISMA flow diagram of selected studies in the qualitative analysis.

A systematic review was conducted following the recommendations of the PRISMA guidelines. A primary search on the PubMed database until December 2017 with the keywords (tDCS AND OCD) yielded 21 results. This primary search was completed by a manual search on articles cited by retrieved articles and on Google allowing for adding five articles (see Figure 1—PRISMA diagram).

The inclusion criteria were: (i) full length original articles published in English language in peer-reviewed journals, (ii) patients with OCD according to DSM or ICD-10 criteria, (iii) detailed description of the stimulation method, and (iv) the use of repeated sessions of tDCS. Among the 26 articles from the primary search, 11 articles were excluded for the following reasons: six were review articles not specifically dealing with tDCS in OCD, four did not concern OCD, and one offered a modelling of the electrical field induced by tDCS in patients with OCD. One of the articles issued by the manual search showed no data on OC symptoms [14], as well as a study investigating the clinical interest of transcranial alternating current stimulation (tACS) and not tDCS [15] were excluded from the qualitative analysis. Another article investigating the effect of a single session of tDCS (anode, cathode, sham) over the medial prefrontal cortex (PFC) on anxiety symptoms after exposure in 12 patients with treatment resistant OCD was also excluded [16].

A total of 12 articles was included in the qualitative analysis, nine from the primary search and three from the manual search [17,18,19].

## 3. Results

Amongst the 12 included studies investigating the clinical effects of tDCS in patients with OCD, eight were case reports [17,19,20,21,22,23,24,25], three were open-label studies, including 5, 8, and 42 patients [18,26,27] and one was a randomized-controlled study including 12 patients with OCD [28]. Remarkably, none of the studies was sham-controlled (Table 1). 

**Figure 2:**
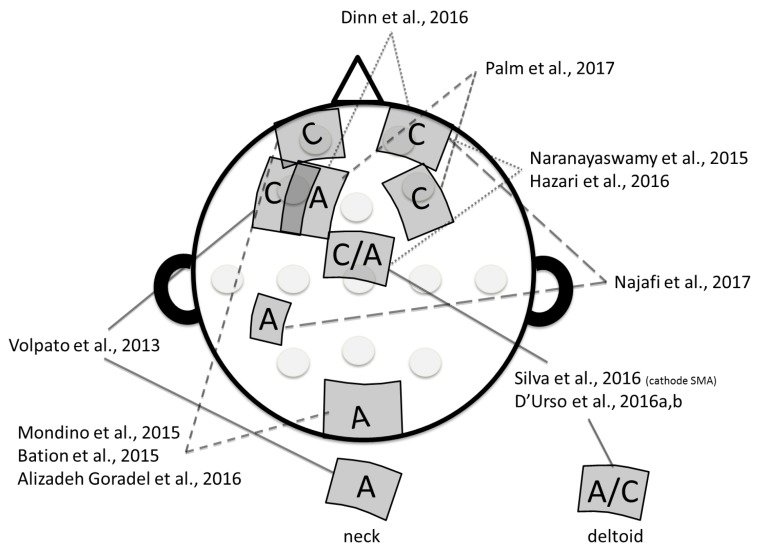
Illustration of the diversity in electrodes montage observed in transcranial direct current stimulation (tDCS) studies aiming to alleviate obsessive-compulsive symptoms in patients with treatment-resistant obsessive-compulsive disorder. A: Anode; C: Cathode. Hazari et al., 2016 [17]; Dinn et al., 2016 [18]; Alizadeh Goradel et al., 2016 [19]; Volpato et al., 2013 [20]; Narayanaswamy et al., 2015 [21]; Mondino et al., 2015 [22]; D’Urso et al., 2016a [23]; Silva et al., 2016 [24]; Palm et al., 2017 [25]; Bation et al., 2015 [26]; Najafi et al., 2017 [27]; D’Urso et al., 2016b [28].

In the first case report, Volpato and colleagues [20] observed no significant effects of 10 sessions of tDCS (20 min, 2 mA) on OC symptoms when the cathode was placed over the left dorsolateral prefrontal cortex (DLPFC; over F3, according to the 10/20 international electroencephalography EEG system) and the anode extra-cephalically (on the neck). Interestingly, the authors reported a significant decrease of depression and anxiety symptoms. Other studies have tried different electrode montages and have shown beneficial outcomes on OC symptoms (see Figure 2 for an illustration of the electrode montages). Namely, two studies targeted the left DLPFC by placing the anode over the left DLPFC (F3) and the cathode either over the right DLPFC (F4) [25] or the right orbitofrontal cortex (OFC) /supraorbital area (Fp2) [18]. Three studies used an electrode montage positioning the cathode over the left OFC (Fp2) and the anode over the occipital region (O2) or the cerebellum [19,22,26]. One study targeted the right OFC (Fp2) with the cathode and the left parieto-temporo-occipital region with the anode (midway between P1, C3, and T7) [27]. Finally, five studies targeted the pre-supplementary motor area (SMA). Among them, two placed the anode over the pre-SMA (Fz/FCz) and the cathode over the right orbitofrontal cortex (Fp2) [17,21], one placed the cathode over the pre-SMA and the anode extra-cephalically over the right deltoid [24], and two compared two different montages with either the anode or the cathode over the pre-SMA and the other electrode extra cephalically over the right deltoid [23,28]. In the included studies, different sizes of electrodes were used: 25 cm^2^ [19,23,24,28], 35 cm^2^ [18,20,21,22,25,26] and 5.5 cm^2^ [27]. The intensity of stimulation was set at 2 mA in all of the tDCS studies (2–3 mA in [27]) and tDCS duration varied from 20 min [17,18,19,20,22,23,26,28] to 30 min [24,25,27]. The number of tDCS sessions also varied; most of the studies delivered 10 [19,20,22,23,26,28] or 20 sessions [17,21,24,25] and one study delivered 15 sessions [18]. tDCS sessions were delivered daily [18,19,23,24,27,28] or twice daily [17,21,22,25,26]. All of the studies used the Yale–Brown Obsessive and Compulsive Scale score (Y-BOCS) [29] to assess OC symptoms.

In summary, a total of 77 patients with OCD received active tDCS with different electrode montages. Most of the studies reported a significant effect of tDCS on OC symptoms, more specifically, a decrease of the YBOCS score. Several studies also reported beneficial effects of tDCS on other symptoms that are often observed in patients with OCD, such as depression and anxiety [18,19,20,24,25].

## 4. Discussion

We reviewed here studies investigating the clinical effects of tDCS in patients with treatment-resistant OCD. Overall, our review included 12 studies, corresponding with a total sample of 77 patients with OCD. Results indicated that applying tDCS might show promising results to reduce OC symptoms. Little is known regarding the duration of this effect since it has not been systematically investigated. Two studies reported that the beneficial effects were still observed at a three-month [27] or seven-month follow-up [17]. In addition, it is interesting to note that some of the included studies also reported beneficial effects of tDCS on depression and anxiety that are common comorbid symptoms in patients with OCD. In line with this, a recent crossover study has investigated the effect of a single session of tDCS on obsession-induced anxiety after symptom provocation in patients with OCD. They reported a significant decrease in the severity of the obsession-induced anxiety following tDCS applied with the cathode over the medial PFC as compared with tDCS applied with the anode over the medial PFC and sham tDCS [16]. One may hypothesize that anxiety, depression, and OCD share abnormalities within brain networks that are targeted by cortical stimulation. However, the findings of beneficial effects of tDCS in OCD should be interpreted with caution and some methodological considerations should be noted.

First, none of the studies that are included in the present review was sham-controlled. One randomized study used a parallel arm design, but compared two active conditions [28]. To the best of our knowledge, only one randomized sham-controlled trial was conducted in OCD patients. This study included 20 patients with OCD and reported that active tDCS (2 mA, 20 min, 15 sessions) applied with the anode over the right DLPFC and the cathode over the left DLPFC improved decision-making abilities as compared to sham tDCS [14]. The authors thus showed that tDCS could have a pro-cognitive effect in patients with OCD, as reported in other psychiatric conditions [5]. However, this study did not provide direct clinical assessment of OC symptoms. Further studies are needed to determine the real effect of repeated sessions of active tDCS on OC symptoms, by comparing with sham. Indeed, previous sham-controlled studies have reported a large sham effect in patients with treatment-resistant OCD receiving repeated sessions of NIBS [11,31].

Second, most of the studies included in our review were case reports and only two studies included more than 10 patients [27,28]. Interpretation of results is thus limited by small sample size. Furthermore, tDCS parameters were highly heterogeneous across studies, in terms of electrode montage (see Figure 2), number of tDCS sessions, tDCS duration, and interval between sessions (from 2 h to 1 day). For instance, regarding electrode positioning, some studies targeted the DLPFC with the anode placed over the left DLPFC (F3) and the cathode over the right OFC [18], or the contralateral DLPFC [25]. Another study placed the cathode over the left DLPFC (F3) and the anode over the neck [20]. Other studies have proposed to target the left OFC (Fp1) or the right OFC (FP2) with the cathode combined with the anode over the right occipital cortex [19,22], the right cerebellum [26] or the temporo-parieto-occipital region [27]. These montages were based on neuroimaging studies showing hyperactivity within the left OFC and hypoactivity within the cerebellum in patients with OCD [9]. The pre-SMA was also commonly targeted in the reviewed studies either with the anode [17,21,23,28] or with the cathode [23,24,28]. In a randomized controlled trial comparing both montages (anode over the pre-SMA or cathode over the pre-SMA), D’Urso and colleagues suggested a better effect of the cathodal-tDCS montage on OC symptoms [28]. Nevertheless, based on these findings, it seems difficult to conclude regarding the optimal tDCS montage to adopt in order to alleviate symptoms in patients with OCD. However, in a computer head modelling study, Senço and colleagues reported interesting findings that may help us identifying the optimal electrode positioning [32]. More precisely, they found that the best theoretical montage to target the neurocircuitry involved in OCD would be with the cathode over the pre-SMA with an extra-cephalic anode, as done in D’Urso and colleagues’ study [28]. 

Regarding the number and duration of tDCS sessions, the choice of delivering 10 to 20 sessions of 20 to 30 min has been mostly extrapolated from the data obtained in studies investigating the clinical effects of tDCS in patients with depression. However, it is not clearly established that increasing the duration and number of sessions leads to a better and longer clinical effect. The interval between consecutive tDCS sessions should also be considered. Indeed, some studies have shown that the inhibitory effects of a session of cathodal tDCS on motor corticospinal excitability were increased if a second tDCS session was performed during the after-effects of the first and were initially reduced and then re-established if the second tDCS session was performed 3 or 24 h after the first one [33]. Furthermore, it was reported in another study that the excitatory effects of anodal tDCS on motor corticospinal excitability were reduced, but prolonged when a second tDCS session was applied during the after-effects of the first (from 0 to 20 min after) but entirely abolished when the second tDCS session was applied 3 or 24 h after the first [34]. 

It is important to mention that most of the patients included in the reviewed studies were treated with different medication (in terms of duration and molecules) when they received tDCS. Most of the patients were treated with SSRIs, but in some studies, they were also treated with other medications, such as serotonin-norepinephrine reuptake inhibitors (SNRI), mood stabilizers or antipsychotics. The concomitant use of medication may influence the effects of tDCS [35]. For instance, studies investigating the effects of tDCS on motor corticospinal excitability have reported that both acute and chronic administration of the SSRI (citalopram) increased and prolonged the excitatory effects that are induced by anodal tDCS and reversed the inhibitory effects of cathodal tDCS into facilitation [36,37]. Furthermore, in a randomized-controlled trial in patients with major depressive disorder, Brunoni et al. have reported that combining tDCS with SSRI (sertraline hydrochloride) induced beneficial clinical improvements that were superior to each treatment taken separately (tDCS only or sertraline only) or sham [38]. Thus, future studies should take into account the concomitant use of medication when investigating the effects of tDCS on OC symptoms.

Clinical characteristics of patients should be taken into account when discussing the role of tDCS in the OCD treatment. For instance, the level of resistance was highly heterogeneous in the reviewed studies; some patients were resistant to several months of combination between SSRI and CBT (e.g., [18,22,23,24,25,26]), some others received ECT [17]. In addition, the subtypes of OCD (obsessions and checking; symmetry and ordering; cleanliness and washing; and, hoarding) [30] were also heterogeneous across studies and might be an important factor to report in future studies. These differences may also account to explain discrepancies observed between studies in term of symptoms improvement (from no effect on OC symptoms [20] to 80% decrease on YBOCS score [17]).

Another limitation is the brain state dependency that may have an impact on the tDCS clinical effect and should also be controlled in future studies. For instance, in the study reporting the largest beneficial effect of tDCS on OC symptoms, patients were not at rest during the stimulation session as in other studies but were required to listen to music and watch movies during the 30-min session duration [27]. In the same way, a single session of cathodal tDCS has been shown to have a beneficial effect when applied during exposure to anxiety [16]. Future studies should investigate the clinical effect of repeated sessions of tDCS when stimulation is applied during exposure to anxiety as compared to with being at rest. 

Finally, up to now, no study has investigated the brain correlates of the symptom improvement following tDCS administration in OCD patients. Investigating biological effects of tDCS in patients with OCD will provide a better understanding of the pathophysiology of OCD (as done with other therapeutics, see [10,11]) and of tDCS’ mechanisms of action. It could be speculated for example that hyperactive cortico-striatal pathways observed in patients with OCD may be down-regulated by either inhibitory stimulation of the OFC or SMA (with the cathode) or by excitatory stimulation (enhancement, with the anode) of the DLPFC. Neuroimaging or electrophysiological investigations are also needed to steer the parameter optimization. In this way, another transcranial electrical stimulation approach has been proposed recently by Klimke and colleagues [15]. In an open-label study including seven patients with OCD, the authors reported the clinical interest of transcranial alternating current stimulation (tACS) applied at gamma frequency (40 Hz). They observed that gamma-tACS applied in a bilateral fronto-temporal montage decreased OC symptoms by 52%, measured by the YBOCS. This novel protocol appears interesting and future studies are needed to further explore the effects of gamma-tACS in patients with OCD. Besides the optimization of stimulation parameters, further studies are also needed to determine the clinical and biological predictors of response, as done in studies on depression [39].

## 5. Conclusions

To conclude, only a few studies investigated the effects of tDCS in OCD, but they showed promising results, with some of them reporting a decrease >35% in YBOCS scores. This effect can be considered as clinically meaningful since the current definition of treatment response is at least a 35% reduction of Y-BOCS score [40]. However, these results are preliminary and further sham-controlled studies are needed to define the role of tDCS in the treatment of OCD and to determine the optimal stimulation parameters to deliver in this indication and subtypes of OCD. To date, regarding the high heterogeneity among studies in terms of the characteristics of patients (e.g., subtypes of OC symptoms, concomitant medication, age) and tDCS parameters (e.g., electrode montage, symptoms provocation paradigm during tDCS), it is difficult to draw a clear conclusion on the efficacy of tDCS in this indication and to propose guidelines for future investigations. Interestingly, based on these preliminary positive reports, randomized clinical trials have been initiated and are now recruiting participants around the world (as seen in clinical trials website: NCT 02407288, 02743715, 03304600). Results from these studies are expected before any conclusion on the relevance of tDCS in patients with OCD can be made. 

## Figures and Tables

**Table 1:** Main findings of studies investigating the clinical interest of transcranial direct current stimulation (tDCS) to decrease symptoms in patients with obsessive-compulsive disorder (OCD).

Articles	N	Patients Characteristics	Target	Intensity Electrode Size	Duration and Number of Sessions	Main Results
Volpato et al., 2013 [20]	1	Age: 35, male	Anode: posterior neck-base Cathode: left DLPFC	2 mA, 35 cm^2^	20 min, 10 sessions (1/day)	No effect on OC symptoms. Depression score decreased (−34% HDRS); anxiety score decreased (−17%).
		Type*: 2,3,4				
		Previous TTT: SSRI, SNRI, CBT				
Mondino et al., 2015 [22]	1	Age: 52, female	Anode: right cerebello-occipital (100 cm^2^) Cathode: left OFC	2 mA, 35 cm^2^	20 min, 10 sessions (2/day; 2 h between 2 sessions)	YBOCS score decreased (−26%)
		Type*: 3,4				
		Previous TTT: tricyclic, SSRI, SNRI, AP, Lithium, CBT				
Hazari et al., 2016 [17]	1	Age: 24, male	Anode: SMA Cathode: right OFC	2 mA, ND	20 min, 20 sessions (2/day, at least 3 h between 2 sessions)	YBOCS decreased (−80%) during 7 months
		Type*: 1,2				
		Previous TTT: SSRI, ECT				
D’Urso et al., 2016 [23]	1	Age: 33, female	Anode: Pre-SMA Cathode: right deltoid And then, Reverse montage	2 mA, 25 cm^2^	20 min, 10 sessions (1/day)	Worsening of symptoms after anodal tDCS.
		Type*: 3				
		Previous TTT: SSRI, BZD, tricyclic, CBT				YBOCS score decreased (−30%) after cathodal tDCS,
Alizadeh Goradel et al., 2016 [19]	1	Age: 23, female	Anode: right occipital Cathode: left OFC	2 mA, 25 cm^2^	20 min, 10 sessions (1/day)	YBOCS score decreased (−64%); Depression score decreased (−87%); −100% anxiety
		Type*: 1				
		Previous TTT: SSRI				
Palm et al., 2017 [25]	1	Age: 31, male	Anode: left DLPFC Cathode: Right DLPFC	2 mA, 35 cm^2^	30 min, 20 sessions (2/day, 3 h between 2 sessions)	Combined with Sertraline, YBOCS score (−22%), depression (−10%) and anxiety (−21%) decreased
		Type*: 1,3				
		Previous TTT: tricyclic, SSRI, AP, CBT				
Narayanaswamy et al., 2015 [21]	2	Age: 39, female	Anode: left pre-SMA Cathode: right OFC	2 mA, 35 cm^2^	20 min, 20 sessions (2/day, at least 3 h between 2 sessions)	Patient 1: YBOCS score decreased (−40%), −52% at day 17
		Type*: 1				
		Previous TTT: SSRI, exposure				
		Age: 24, male				Patient 2: YBOCS score decreased (−46.7%)
		Type*: 1				
		Previous TTT: tricyclic, SSRI				
Silva et al., 2016 [24]	2	Age: 37, male	Anode: right deltoid Cathode: bilateral SMA	2 mA, 25 cm^2^	30 min, 20 sessions (1/day)	Patient 1: no effect at Week 4, YBOCS score decreased at week 12 (−18%). No changes in anxiety nor depression
		Type*: 2				
		Previous TTT: tricyclic, SSRI, CBT				
		Age: 31, male				Patient 2: YBOCS score decreased, (−17%) at Week 4; −55% at week 12). 50% improvement in anxiety and depression
		Type*: 1,3				
		Previous TTT: tricyclic, SSRI				
Dinn et al., 2016 [18]	5	Age: 40.4 (8.4), 4 females, 1 male	Anode: left DLPFC Cathode: right OFC	2 mA, 35 cm^2^	20 min, 15 sessions (1/day)	Open Label Study
		Type*: ND				
		Previous TTT: SSRI, SNRI, AP				OC symptoms decreased (−23%); depression decreased (−30%)
Bation et al., 2015 [26]	8	Age: 44.2 (13.8), 6 females, 2 males	Anode: right cerebellum Cathode: left OFC	2 mA, 35 cm^2^	20 min, 10 sessions (2/day, at least 3 h between 2 sessions)	Open Label Study
		Type*: 1 (*n* = 5), 3 (*n* = 3)				
		Previous TTT: tricyclic, 3 SSRI, AP, CBT				YBOCS score decreased (−24.6%)
D’Urso et al., 2016 [28]	12	Age: 39.0 (13.1), 7 females, 5 males	Anode midline pre SMA Cathode: right deltoid (*n* = 6) OR reverse montage	2 mA, 25 cm^2^	20 min, 10 sessions (1/day)	RCT—10 patients completed the study
		Type*: 1 (*n* = 4), 2 (*n* = 2), 3 (*n* = 6)				Cathodal tDCS was significantly more effective than anodal tDCS. In cathodal arm, YBOCS score decreased (−17.5%) after 10 sessions, −20.1% after 20 sessions
		Previous TTT: at least SSRI, CBT				
Najafi et al., 2017 [27]	42	Age: 29.1 (10.1), 23 females, 19 males	Anode: parieto-temporo-occipital areas Cathode: right OFC	2–3 mA, 5.5 cm²	30 min, 15 sessions (1/day)	Open Label Study
						YBOCS score decreased (−63.4%)
		Type*: ND				
		Previous TTT: at least 2 SSRI, CBT				Maintenance of the effect at 3 months follow up (−77.6%)

DLPFC: dorsolateral prefrontal cortex; HDRS: Hamilton Depression Rating Scale; ND: Not Done; OFC: orbitofrontal cortex; (pre) SMA: (pre) supplementary motor area; Y-BOCS: Yale-Brown Obsessive Compulsive Scale. Age: mean (standard deviation) years, TTT: treatment, AP: antipsychotic, SSRI: selective serotonin reuptake inhibitor, SNRI: serotonin–norepinephrine reuptake inhibitor, CBT: cognitive behavioural therapy. Type* 1 = obsessions and checking, 2 = symmetry and ordering, 3 = cleanliness and washing, 4 = hoarding according to Leckman et al. 1997 [30].
